# Association of the EHRA/HRS/APHRS/LHRS definition of atrial cardiomyopathy with incident atrial fibrillation, heart failure, and stroke

**DOI:** 10.1093/europace/euag259

**Published:** 2026-09-29

**Authors:** Khalid Kazi, Ali Wahab, Catherine Revnolds, Gregory Y H Lip, Jianhua Wu, Andreas Goette, Chris P Gale, Ramesh Nadarajah

**Affiliations:** Leeds Institute of Data Analytics, University of Leeds, Leeds, UK; Leeds Institute for Cardiovascular and Metabolic Medicine, University of Leeds, Leeds, UK; Department of Cardiology, Leeds Teaching Hospitals NHS Trust, Leeds, UK; Department of Cardiology, Leeds Teaching Hospitals NHS Trust, Leeds, UK; Leeds Institute for Cardiovascular and Metabolic Medicine, University of Leeds, Leeds, UK; Liverpool Centre for Cardiovascular Science at University of Liverpool, Liverpool John Moores University and Liverpool Heart & Chest Hospital, Liverpool, UK; Department of Clinical Medicine, Danish Center for Health Services Research, Aalborg University, Aalborg, Denmark; Medical University of Bialystok, Bialystok, Poland; Wolfson Institute of Population Health, Queen Mary, University of London, London, UK; Department of Cardiology and Intensive Care Medicine, St. Vincenz-Hospital Paderborn, Am Busdorf 2, Paderborn 33098, Germany; MAESTRIA Consortium at AFNET, Münster, Germany; Medical Faculty, Otto-von-Guericke University, Magdeburg, Germany; Leeds Institute of Data Analytics, University of Leeds, Leeds, UK; Leeds Institute for Cardiovascular and Metabolic Medicine, University of Leeds, Leeds, UK; Department of Cardiology, Leeds Teaching Hospitals NHS Trust, Leeds, UK; Leeds Institute of Data Analytics, University of Leeds, Leeds, UK; Leeds Institute for Cardiovascular and Metabolic Medicine, University of Leeds, Leeds, UK; Department of Cardiology, Leeds Teaching Hospitals NHS Trust, Leeds, UK

**Keywords:** Atrial cardiomyopathy, Stroke, Heart failure, Atrial fibrillation, Prognosis

Atrial cardiomyopathy (AtCM) is defined, in a clinical consensus statement from the European Heart Rhythm Association (EHRA), Heart Rhythm Society (HRS), Asian Pacific Heart Rhythm Society (APHRS) and Latin American Heart Rhythm Society (LHRS), as any complex of structural, architectural, contractile, or electrophysiological changes affecting the atria with the potential to produce clinically relevant manifestations.^1^ AtCM is a distinct clinical entity and a potential final common pathway for atrial fibrillation (AF), heart failure (HF), and stroke.^2^ An outstanding knowledge gap from the EHRA/HRS/APHRS/LHRS consensus statement was the threshold of AtCM that portends increased risk.^1^ To address this, we conducted a prospective study to investigate rates of incident AF, HF, and stroke in those with and without AtCM.

The protocol for the FIND-AF longitudinal cohort study has been published previously.^3^ The study has ethical approval (North West—Greater Manchester South Research Ethics Committee reference 23/NW/0180). FIND-AF is a machine learning algorithm that uses routine electronic health record (EHR) data to stratify risk for incident AF diagnosis within the next 6 months.^4–7^ Patients at high FIND-AF risk, without AF found during ECG screening, were recruited to undergo transthoracic echocardiography, biomarker sampling, and electrocardiographic analysis of lead I ECGs (*n* = 296). Patients with prior history of AF, HF, or stroke were excluded from this analysis (*n* = 65). We applied the EHRA/HRS/APHRS/LHRS 2024 definition of AtCM, including abnormality of atrial geometry (indexed left atrial volume ≥34 mL/m^2^), electrophysiology (PR interval ≥200 ms),^8^ and serum biomarker (NT-proBNP >365 pg/mL). We excluded participants with incomplete data (*n* = 20). AtCM was considered present if one of the three domains was met, with three stages (Stage 1–3 AtCM) defined by the proportion of domains met. The primary outcome was a composite of incident AF, HF, or stroke, with follow-up through routine EHRs. Baseline characteristics were compared by AtCM status. Cumulative incidence of the primary outcome is shown with Kaplan–Meier curves and log-rank tests, and hazard ratios estimated from Cox proportional-hazards models adjusted for age, hypertension, and diabetes. We also conducted a sensitivity analysis replacing the PR interval for *P*-wave duration ≥120 ms. Statistical significance was set at *P* < 0.05.

Of 211 participants, median age was 75.0 years (interquartile range [IQR] 72.0–78.0), with 109 (51.7%) women; 130 (61.6%) of participants met the EHRA/HRS/APHRS/LHRS criteria for AtCM. Participants with AtCM were older than those without (75.0, IQR 72.0–79.0 vs. 75.0, IQR 70.0–77.0, *P* = 0.04), but there was no statistical difference between groups for sex distribution or prevalence of hypertension, diabetes, ischaemic heart disease, or chronic kidney disease.

Over median follow up 24.5 (IQR 20.2–28.3) months, 16/130 participants with AtCM experienced a primary outcome (6.5 events per 100 person-years) compared with 1/81 participants without AtCM (0.6 events per 100 person-years) (log rank test *P* = 0.005, adjusted hazard ratio [aHR] 9.3, 95% confidence interval [CI] 1.2–70.4, *P* = 0.03) (*Figure 1A*).

**Figure 1:**
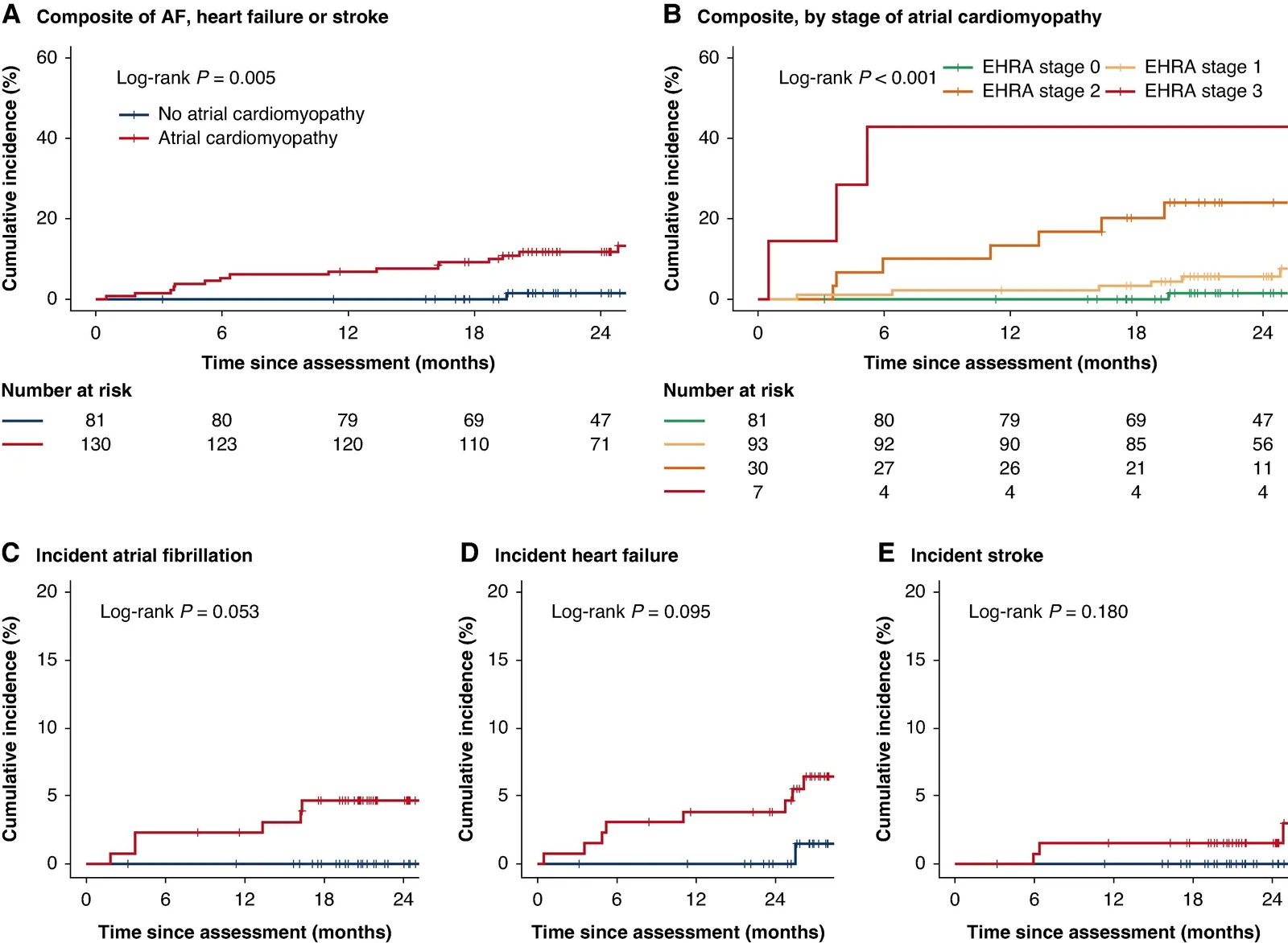
Cumulative incidence of the primary outcome and its components by atrial cardiomyopathy status. (*A*) Composite of incident atrial fibrillation, heart failure or stroke, by AtCM status. (*B*) Composite, by stage of AtCM. (*C*) Incident atrial fibrillation, (*D*) incident heart failure and (*E*) incident stroke, each by AtCM status.

Of 130 participants with AtCM, 93 (71.5%) had stage 1 AtCM, 30 (23.1%) had Stage 2 AtCM, and 7 (5.4%) had stage 3 AtCM. Primary outcome events were experienced by 6/93 participants with Stage 1 AtCM (3.2 events per 100 person-years), 7/30 participants with Stage 2 AtCM (13.7 per 100 person-years), and 3/7 participants with Stage 3 AtCM (30.2 events per 100 person-years). Compared to participants without AtCM the aHRs for the primary outcome for Stage 1, Stage 2, and Stage 3 AtCM were 4.9 (95% CI 0.6–41.0, *P* = 0.14), 22.1 (2.6–187.1, *P* < 0.01), and 45.7 (4.6–456.4, *P* < 0.01), respectively (*Figure 1B*). Per increase in stage of AtCM the aHR increased by 3.5 times (95% CI 2.0–6.0, *P* < 0.01). Sensitivity analyses did not affect the size or magnitude of effect. The composite comprised 10 incident heart failure, 6 incident AF, and 3 incident ischaemic stroke events, all AF and stroke events occurring in participants with AtCM (*Figure 1C–E*). Component-specific hazard ratios were not estimable, as no AF or stroke events occurred in participants without AtCM. No stroke was preceded by an incident AF diagnosis.

This hypothesis-generating data from a well-characterized cohort of older and comorbid patients, who were well-matched between those with and without AtCM, suggest that the EHRA/HRS/APHRS/LHRS 2024 consensus definition of AtCM has prognostic relevance, with AtCM of Stage 2 severity or greater particularly associated with increased risk of incident AF, HF, and stroke.

Our findings build upon UK Biobank data suggesting that AtCM, in those without AF, HF, or stroke at baseline, is associated with increased risk of these outcomes.^2^ In both studies, ≥2 AtCM markers conferred increased risk, suggesting preventive efforts may be most beneficial from Stage 2 AtCM onwards. However, that study used markers (*P*-wave terminal force and left atrial emptying fraction on cardiac magnetic resonance imaging)^2^ less feasible to apply at scale, whereas the EHRA/HRS/APHRS/LHRS definition, incorporating echocardiographic left atrial dilatation, PR interval prolongation (or P-wave duration) and NT-proBNP, can readily be implemented clinically. This cohort was a decade older, with substantially higher event rates. Prospective assessment is now required to determine whether intervention can prevent these outcomes, and an older comorbid population may make such a trial more feasible. Amongst high FIND-AF risk participants, almost two-thirds had AtCM, suggesting this may be a viable recruitment tool.

We recognize the limitations of this study. First, this is a cohort from a single country, and whether the prognostic associations of AtCM generalize internationally requires further study. Second, the number of incident events was small, which precluded component-specific modelling and produced wide CIs; the magnitude of association should be regarded as imprecise, although its direction and the stage gradient were consistent across models. Longer follow-up is ongoing. Third, we could not compare the EHRA/HRS/APHRS/LHRS definition to the Heart Failure Association of the European Society of Cardiology definition,^9^ as this pragmatic study did not include left atrial reservoir strain. Fourth, without continuous rhythm monitoring, we could not assess AF burden, which extends beyond the dichotomous presence or absence of AF and may relate more closely to outcomes.^10^

In summary, this prospective cohort study suggests that the EHRA/HRS/APHRS/LHRS 2024 consensus definition of AtCM is associated with incident AF, HF, and stroke and may represent a treatment target.

## Data Availability

Data are available on reasonable request. Technical appendix, statistical code and dataset are available from the corresponding author at r.nadarajah@leeds.ac.uk.
